# Admission white blood cell count predicts post-discharge mortality in patients with acute aortic dissection: data from the MIMIC-III database

**DOI:** 10.1186/s12872-021-02275-0

**Published:** 2021-09-25

**Authors:** Chiyuan Zhang, Zuli Fu, Hui Bai, Guoqiang Lin, Ruizheng Shi, Xuliang Chen, Qian Xu

**Affiliations:** 1 Department of Cardiology, The Third Xiangya Hospital, Central South University, Changsha, Hunan China; 2 Department of Cardiovascular Medicine, Xiangya Hospital, Central South University, Changsha, Hunan China; 3 Department of Cardiovascular Surgery, Xiangya Hospital, Central South University, Xiangya Rd 87, Changsha, 410008 Hunan China

**Keywords:** White blood cell count, Acute aortic dissection, Post-discharge mortality, MIMIC-III database

## Abstract

**Background:**

Inflammation underlies both the pathogenesis and prognosis in patients with acute aortic dissection (AAD). This study aimed to assess the association of ICU admission of white blood cell count (WBCc) with post-discharge mortality in these patients.

**Methods:**

Clinical data were extracted from the MIMIC-III V1.4 database. After adjusted to covariables, Cox regression analysis and Kaplan–Meier survival curve were performed to determine the relationship between WBCc on admission and post-discharge mortality (30-day, 90-day, 1-year and 5-year) in AAD patients. Subgroup analysis and receiver operating characteristic (ROC) curve analysis were used to test the performance of WBCc in predicting mortality in AAD patients.

**Results:**

A total of 325 eligible patients were divided into 2 groups: normal-WBCc group (≤ 11 k/uL) and high-WBCc group (> 11 K/uL). In univariate Cox regression analysis, high WBCc was significant risk predictor of 30-day, 90-day, 1-year and 5-year mortality [hazard ratio (HR), 95% CI, *P* 2.58 1.36–4.91 0.004; 3.16 1.76–5.70 0.000; 2.74 1.57–4.79 0.000; 2.10 1.23–3.54 0.006]. After adjusting for age and other risks, high WBCc remained a significant predictor of 30-day, 90-day and 1-year mortality in AAD patients (HR, 95% CI, *P* 1.994 1.058–3.76 0.033; 2.118 1.175–3.819 0.013; 2.37 1.343–4.181 0.003). The area under ROC curve of WBCc for predicting 30-day, 90-day, 1-year and 5-year mortality were 0.69, 0.70, 0.66 and 0.61, respectively. The results from subgroups analysis showed that there was no interaction in most strata and patients who were younger than 69 years of age or had history of respiratory disease with an elevated WBCc had an excess risk of 30-day mortality (HR, 95% CI, *P* 3.18 1.41–7.14 0.005; 3.84 1.05–14.13 0.043).

**Conclusions:**

Higher than normal WBCc on admission may predict post-discharge mortality in patients with AAD.

## Background

Acute aortic dissection (AAD) is a devastating cardiovascular disease with urgent onset, rapid progression, and high mortality [1]. Statistics figures showed that the mortality rate of AAD was an increase of 1–2% per hour after the onset of symptoms [2], and was ranged from 36 to 72% in the intensive care unit (ICU) during first 48 h [3]. Thus, it is of great significance for risk stratification and management to evaluate the risks factors for clinical outcomes in AAD patients.

Inflammation is involved in the occurrence and development of AAD [4, 5]. In recent years, some studies showed that inflammatory reactants such as D-dimer [6], C-reactive protein (CRP) [7], platelet count (PLTc) [8] and fibrinogen [9] were associated with the clinical outcomes of AAD patients, but these results are controversial and need to be further verified in larger population and longer follow-up time. White blood cell count (WBCc) is a common clinical biochemical index to reflect acute inflammation, which has been used to detect vascular inflammation and predict cardiovascular risk [10]. Recently, elevated WBCc on admission was found to be related to increased in-hospital death in patents with AAD [11, 12]. However, the relationship between it and post-discharge mortality were poorly defined. This study aimed to evaluate and analyze the prognostic of WBCc on post-discharge mortality among AAD patients.

## Methods

This was a retrospective study based on a publicly available Medical Information Mart for Intensive Care (MIMIC) III database. It is a large, single-center database containing comprehensive medical information for more than 60,000 ICU admissions at Beth Israel Deaconess Medical Center (BIDMC) in Boston, Massachusetts from 2001 to 2012 [13]. MIMIC-III data are Health Insurance Portability and Accountability Act of 1996 (HIPAA) compliant, and all investigators with data access (MEG, RG) were approved by PhysioNet. Information available in MIMIC-III includes general information (i.e., demographics, insurance, ethnicity, etc.), treatment process (i.e., charted clinical observations, laboratory tests, physiological scores, medications, surgery, etc.) and survival data.

**Fig. 1:**
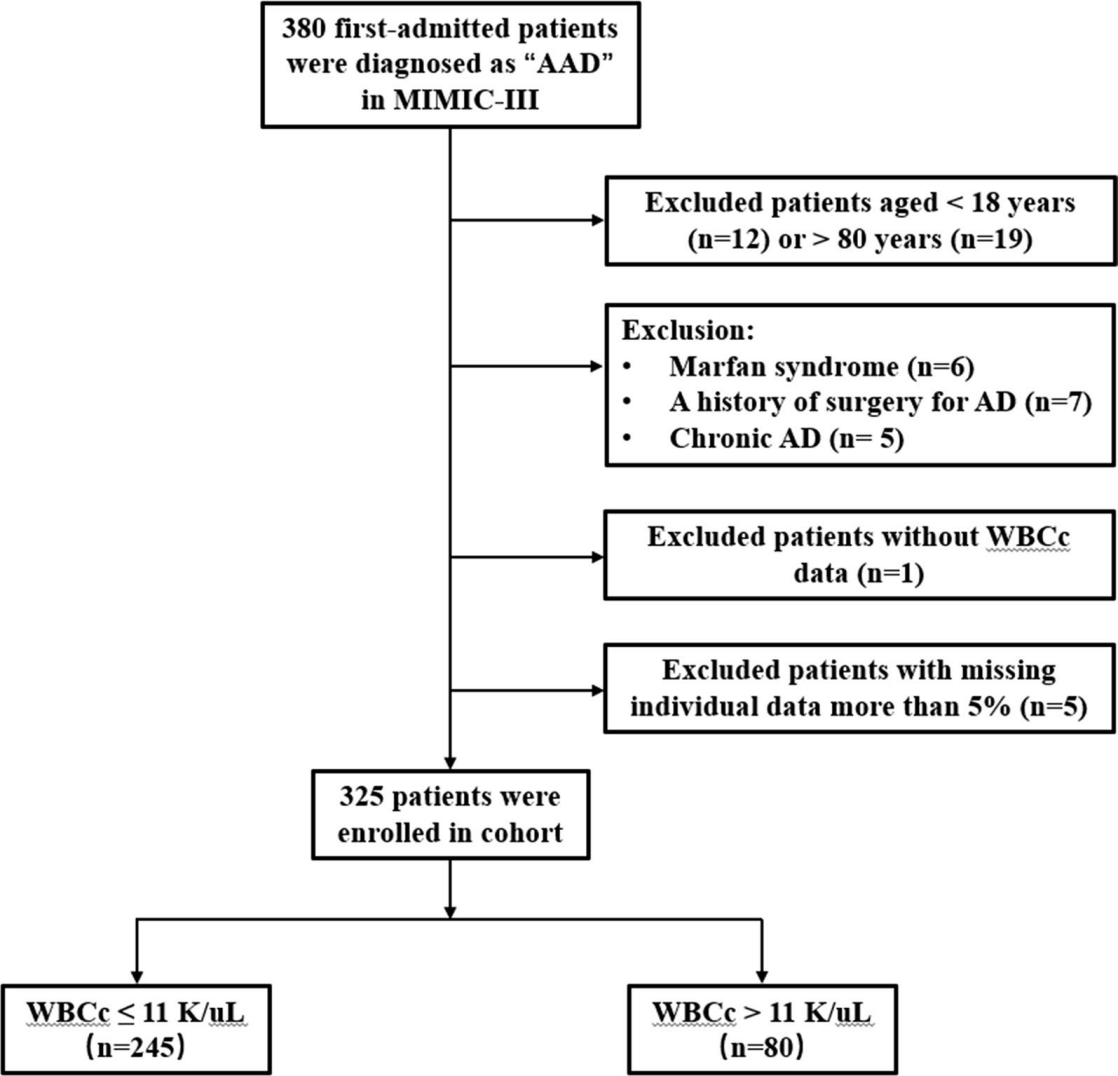
Study flow chart in the present study. AAD: acute aortic dissection; AD: aortic dissection; WBCc: white blood cell count

We included patients with AAD including both Stanford type A and type B based on the International Classification of Diseases 9th Edition (ICD-9) code in MIMIC-III database. Of these patients, we excluded those including: (1) For the adult study and avoiding more confounding factors, patients aged < 18 years or > 80 years were excluded; (2) patients who had a clear etiology, such as Marfan syndrome, iatrogenic aortic dissection (AD) secondary to cardiac surgery, a history of surgery for AD, or chronic AD; (3) no WBCc data; (4) missing individual data including demographics, laboratory tests, comorbidities, etc. more than 5%. Enrolled AAD patients were divided into 2 groups according to the admission WBCc > 11 K/uL and ≤ 11 K/uL as a cut-off value for normal. The complete process was shown in Fig. 1.

Data extraction was performed through Structured Query Language (SQL) with PostgreSQL 9.6. Baseline characteristics after ICU admission were collected, including demographics (age, gender, ethnicity, etc.), vital signs, laboratory tests, comorbidities, severity score and other data. Vital signs included systolic blood pressure (SBP), diastolic blood pressure (DBP), and mean blood pressure (MBP). The laboratory parameters including red blood cell (RBC), red cell distribution width (RDW), hemoglobin (HB), hematocrit (HCT), platelet (PLT); activated partial thromboplastin time (APTT), prothrombin tmie (PT), international normalized ratio (INR); blood urea nitrogen (BUN), Creatinine (Cr); glucose; serum potassium, sodium, chlorine, magnesium, and total calcium were measured during the admission. Comorbidities including hypertension; diabetes; hypercholesterolemia (HC); valvular disease; stroke; coronary artery disease (CAD); congestive heart failure (CHF); atrial fibrillation (AF); liver disease; respiratory disease, acute kidney injury (AKI) and renal replacement therapy (RRT) were also collected for analysis based on the recorded ICD-9 codes in the MIMIC-III database. Severity score included sequential organ failure assessment (SOFA) score; simplified acute physiology score (SAPS II); oxford acute severity of illness score (OASIS) and systemic inflammatory response syndrome (SIRS) score.

Shapiro–Wilk tests were used to examine whether the continuous variables conform to the normal distribution. Normally distributed continuous variables were presented as the mean ± SD and non-normally distributed continuous variables were presented as the median and interquartile range (IQR). Categorical variables were presented by number and percentage. Continuous data were compared using Student *t* test or Mann–Whitney U test and categorical data were compared using chi-squared test as appropriate. Survival rates within normal-WBCc and high-WBCc groups were compared by the Kaplan–Meier curve and the log-rank tests. Cox regression models were conducted to evaluate the predictive value of WBCc in post-discharge including 30-day, 90-day, 1-year and 5-year mortality with hazard ratios (HRs) and 95% confidence intervals (CIs). Subgroup analyses were conducted to evaluate the WBCc and 30-day morality in different subgroups, including gender; age; hypertension; diabetes; HC; valvular disease; CHF; AF; liver disease; respiratory disease; AKI and RRT. ROC curve analyses and calculation of AUC were used to examine the performance of WBCc in predicting morality. A *P* value < 0.05 was considered statistically significant. All of the statistical analyses were performed by the EmpowerStats ver 2.17.8 (http://www.empowerstats.com/cn/, X&Y solutions, Inc., Boston, MA) and R software vers 3.42. The raw data showed in this study are fully available in MIMIC-III database.

## Results

After reviewing the data of 380 AAD patients, a total of 325 eligible patients were enrolled in this study (detailed flow chart of patients’ selection shown in Fig. 1). The baseline characteristics of all patients are summarized in Table 1. The mean age of all patients was 68.0 (55.4–77.2) years, and 63.1% of patients (205/325) were male. According to admission WBCc, patients were divided into 2 groups including normal-WBCc group and high-WBCc group (≤ 11 K/uL; > 11 K/uL). Patients with an elevated WBCc had higher PLT, HCT, Hb, BUN and Glucose. Additionally, these patients had more CHF and higher SAPS II and SIRS scores (all *P* < 0.05).

**Table 1:** Baseline characteristics of patients

Characteristics	White blood cell count (K/uL)		*P* value
	≤ 11 (n = 245)	> 11 (n = 80)	
Demographics			
Gender			0.145
Male	160 (65.3%)	45 (56.3%)	
Female	85 (34.7%)	35 (43.8%)	
Age, years	69.0 (55.0–77.0)	64.5 (55.0–77.0)	0.378
Insurance			0.786
Government	16 (6.5%)	4 (5.0%)	
Medicaid	11 (4.5%)	4 (5.0%)	
Medicare	130 (53.1%)	41 (51.3%)	
Private	83 (33.9%)	31 (38.8%)	
Selfpay	5 (2.0%)	0 (0.0%)	
Ethnicity			0.846
White	166 (67.8%)	56 (70.0%)	
Black	32 (13.1%)	12 (15.0%)	
Hispanic	11 (4.5%)	2 (2.5%)	
Others	36 (14.7%)	10 (12.5%)	
Dissection site			0.152
Thoracic	143 (58.4%)	38 (47.5%)	
Abdominal	38 (15.5%)	19 (23.8%)	
Thoracoabdominal	64 (26.1%)	23 (28.8%)	
Sanford type			0.058
A	172 (70.2%)	47 (58.7%)	
B	73 (29.8%)	33 (41.3%)	
Comorbidities			
Hypertension	142 (58.0%)	50 (62.5%)	0.473
Diabetes	19 (7.8%)	8 (10.0%)	0.528
HC	38 (15.5%)	12 (15.0%)	0.913
Valvular disease	22 (9.0%)	11 (13.8%)	0.220
Stroke	18 (7.3%)	7 (8.8%)	0.683
CAD	44 (18.0%)	14 (17.5%)	0.926
CHF	11 (4.5%)	18 (22.5%)	0.001
Atrial fibrillation	42 (17.1%)	14 (17.5%)	0.941
Renal disease	30 (12.2%)	15 (18.8%)	0.144
Liver disease	9 (3.7%)	1 (1.3%)	0.461
Respiratory disease	49 (20.0%)	14 (17.5%)	0.623
AKI	96 (39.2%)	37 (46.3%)	0.264
RRT	20 (8.2%)	9 (11.3%)	0.400
Laboratory test			
RBC, K/uL	3.7 ± 0.6	3.8 ± 0.9	0.234
PLT, K/uL	154.0 (116.0–220.0)	180.5 (136.5–265.5)	0.021
RDW, %	14.4 (13.6–15.4)	14.6 (13.7–15.3)	0.449
HCT, %	28.0 (22.0–33.0)	30.4 ± 6.9	0.006
Hb, g/dL	9.6 (7.6–11.5)	10.3 ± 2.4	0.029
BUN, mg/dL	15.0 (12.0–20.0)	20.0 (15.0–26.5)	< 0.001
Creatinine, mg/dL	1.0 (1.0–2.0)	1.0 (1.0–2.0)	0.064
Chloride, mmol/L	102.0 (100.0–105.0)	102.4 ± 4.2	0.899
Glucose, mg/dL	100.0 (88.0–116.0)	110.5 (91.0–126.0)	0.013
APTT, s	28.4 (25.4–32.7)	27.7 (24.4–33.0)	0.571
INR	1.0 (1.0–1.0)	1.0 (1.0–1.0)	0.148
PT, s	13.4 (12.6–14.4)	13.4 (12.4–14.8)	0.769
Sodium, mmol/L	136.0 (134.0–139.0)	136.6 ± 3.3	0.246
Potassium, mmol/L	3.6 (3.3–3.9)	3.7 ± 0.6	0.187
Total Ca2, mg/dL	9.0 (8.0–9.0)	8.5 (8.0–9.0)	0.618
Magnesium, mg/dL	2.0 (1.9–2.3)	2.0 (1.9–2.3)	0.633
SBP, mmHg	151.0 (136.0–164.0)	149.5 (134.5–164.5)	0.859
DBP, mmHg	80.0 (72.0–89.0)	82.0 (71.0–93.0)	0.504
MAP, mmHg	102.0 (93.0–111.0)	103.0 (92.5–114.0)	0.590
Severity score			
SAPSII	34.0 (27.0–41.0)	40.5 ± 14.3	0.003
OASIS	32.0 (27.0–37.0)	34.0 (26.0–41.5)	0.173
SOFA	4.0 (2.0–6.0)	4.0 (2.0–7.0)	0.624
SIRS	2.0 (2.0–3.0)	3.0 (3.0–4.0)	< 0.001
Treatment			0.366
Medical	58 (31.0%)	22 (28.6%)	
Surgical	84 (44.9%)	30 (39.0%)	
Endovascular	45 (24.1%)	25 (32.5%)	

Data are presented as mean ± SD, n (%), or medians (interquartile ranges). HC: hypercholesterolemia; CAD: coronary artery disease; CHF: congestive heart failure; AKI: acute kidney injury; RRT: renal replacement therapy; RBC: red blood cell; PLT: platelet; RDW: red cell distribution width; Hb: hemoglobin; HCT: hematocrit; BUN: blood urea nitrogen; APTT: activated partial thromboplastin time; PT: prothrombin time; INR: international normalized ratio; Total Ca: total calcium; SBP: systolic blood pressure; DBP: diastolic blood pressure; MBP: mean blood pressure; SAPS II: simplified acute physiology score; OASIS: oxford acute severity of illness score; SOFA: sequential organ failure assessment; SIRS: systemic inflammatory response syndrome

**Fig. 2:**
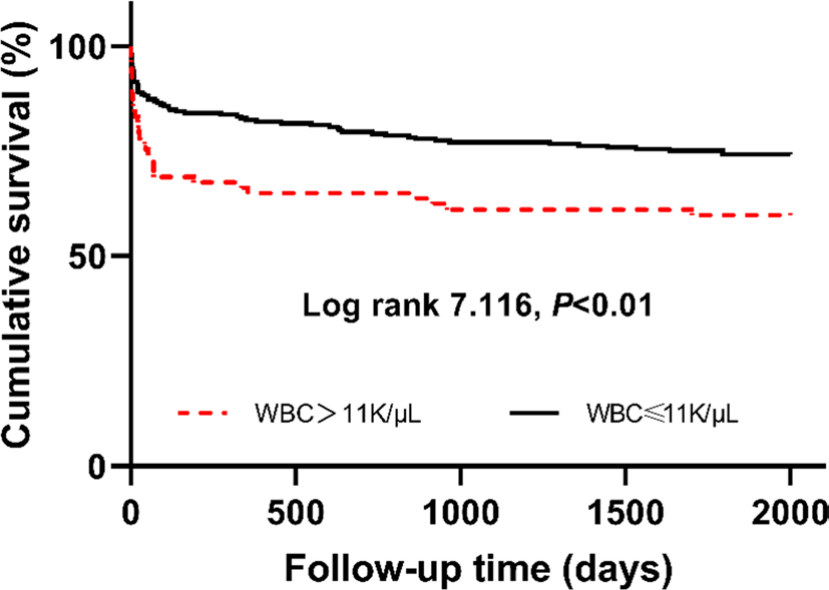
Kaplan–Meier survival curve for post-discharge survival stratified by admission white blood cell count. Survival rate were lower in high-WBCc group (log-rank *P* < 0.01); WBCc: white blood cell count

During the 5-year follow-up, 98 patients died. The overall 30-, 90-day and 1-, 5-year mortality rate were 14.8% (48/325), 18.8% (61/325), 22.8% (74/325) and 30.2% (98/325), respectively. In Fig. 2, the Kaplan–Meier survival curve indicated that the survival rate of high-WBCc group was significantly lower than normal-WBCc group during these periods (log-rank *P* < 0.01).

In order to explore the association between admission WBCc and post-discharge mortality, Cox regression analysis was performed and listed in Table 2. In the univariate Cox regression analysis, compared with the referent group (normal-WBCc: ≤ 11 K/uL), high WBCc was a significant predictor of 30-day, 90-day, 1-year and 5-year mortality in patients with AAD (HR, 95% CI, *P* 2.58 1.36–4.91 0.004; 3.16 1.76–5.70 0.000; 2.74 1.57–4.79 0.000; 2.10 1.23–3.54 0.006). In the multivariate Cox regression analysis, after adjusting to age, hypertension, valvular disease, stroke, CHF, atrial fibrillation, renal disease, BUN, type of AAD and treatment type, high-WBCc remained a significant predictor of 30-day, 90-day and 1-year mortality in AAD patients (HR, 95% CI, *P* 1.994 1.058–3.76 0.033; 2.118 1.175–3.819 0.013; 2.37 1.343–4.181 0.003), but not a predictor of 5-year mortality (HR: 1.499, 95% CI 0.932–2.411, *P* 0.095). Moreover, as shown in Table 3, the AUC of level in predicting 30-day, 90-day, 1-year and 5-year mortality were 0.69, 0.70, 0.66 and 0.61, respectively. Compared with other classic severity scores, WBCc showed a better performance than SIRS score.

**Table 2:** Hazard Ratio (HR) (95% CIs) for mortality across groups of admission WBCc

WBCc	Non-adjusted		Model 1	
	HR (95% CIs)	*P* value	HR (95% CIs)	*P* value
30-day mortality WBCc, K/uL				
≤ 11 K/uL	1.0 (ref)	/	1.0 (ref)	/
> 11 K/uL	2.583 (1.361–4.905)	0.004	1.994 (1.058–3.76)	0.033
90-day mortality WBCc, K/uL				
≤ 11 K/uL	1.0 (ref)	/	1.0 (ref)	/
> 11 K/uL	3.161 (1.756–5.693)	< 0.001	2.118(1.175–3.819)	0.013
1-year mortality WBCc, K/uL				
≤ 11 K/uL	1.0 (ref)	/	1.0 (ref)	/
> 11 K/uL	2.741 (1.569–4.789)	< 0.001	2.37 (1.343–4.181)	0.003
5-year mortality WBCc, K/uL				
≤ 11 K/uL	1.0 (ref)	/	1.0 (ref)	/
> 11 K/uL	2.090 (1.234–3.541)	0.006	1.499 (0.932–2.411)	0.095

Non-adjusted model adjusted to: none;

Adjusted model 1 adjusted to: age, hypertension, valvular disease, stroke, CHF, atrial fibrillation, renal disease, BUN, type of AAD and treatment type

HR: Hazard Ratio; WBCc: White blood cell count; CHF: congestive heart failure; BUN: blood urea nitrogen

**Table 3:** Area under receiver operating characteristic (ROC) curve of WBCc and severity scores

Terms	AUC
30-day mortality	
WBC	0.691
SAPSII	0.796
OASIS	0.812
SOFA	0.697
SIRS	0.648
90-day mortality	
WBC	0.697
SAPSII	0.779
OASIS	0.786
SOFA	0.716
SIRS	0.644
1-year mortality	
WBC	0.659
SAPSII	0.745
OASIS	0.755
SOFA	0.677
SIRS	0.622
5-year mortality	
WBC	0.610
SAPSII	0.701
OASIS	0.685
SOFA	0.626
SIRS	0.585

AUC: area under curve; *P* < 0.05

For further analysis, patients were divided into different subgroups (gender, age, hypertension, diabetes, HC, valvular disease, CHF, AF, liver disease, respiratory disease, AKI and RRT). As shown in Table 4, the results showed that there was no interaction in most strata (*P* for interaction = 0.13–1.00). Patients who were younger than 69 years of age or had a history of respiratory disease with an elevated WBCc had an excess risk of 30-day mortality (HR, 95% CI, *P* 3.18 1.41–7.14 0.005; 3.84 1.05–14.13 0.043).

**Table 4:** Subgroup analysis of the association with admission WBCc and 30-day mortality

	Number of patients	WBCc			*P* for interaction
		≤ 11 K/uL	*P* value	> 11 K/uL	
Gender					0.676
Male	205	ref	0.001	3.799 (1.695–8.513)	
Female	120	ref	0.550	1.392 (0.471–4.113)	
Age					0.013
< 69	167	ref	0.005	3.175 (1.412–7.141)	
≥ 69	158	ref	0.405	1.609 (0.525–4.934)	
Hypertension					0.260
No	133	ref	0.017	3.179 (1.235–8.181)	
Yes	192	ref	0.067	2.286 (0.943–5.543)	
Diabetes					0.085
No	298	ref	0.013	2.378 (1.204–4.694)	
Yes	27	ref	0.119	5.100 (0.658–39.548)	
HC					0.529
No	275	ref	0.001	3.261 (1.636–6.500)	
Yes	50	ref	0.524	0.485 (0.052–4.487)	
Valvular disease					0.246
No	292	ref	0.003	2.843 (1.416–5.709)	
Yes	33	ref	0.774	1.275 (0.242–6.704)	
CHF					0.119
No	296	ref	0.017	2.348 (1.163–4.741)	
Yes	29	ref	0.999	/	
AF					0.148
No	269	ref	0.011	2.504 (1.230–5.098)	
Yes	56	ref	0.153	2.960 (0.668–13.118)	
Liver disease					1.000
No	315	ref	0.002	2.738 (1.429–5.248)	
Yes	10	ref	1.000	0.000	
Respiratory disease					0.005
No	262	ref	0.024	2.369 (1.119–5.015)	
Yes	63	ref	0.043	3.844 (1.046–14.127)	
AKI					0.216
No	192	ref	0.004	3.458 (1.473–8.121)	
Yes	133	ref	0.256	1.761 (0.663–4.679)	
RRT					0.129
No	296	ref	0.008	2.519 (1.269–4.999)	
Yes	29	ref	0.270	2.833 (0.445–18.042)	

WBCc: White blood cell count; HC: hypercholesterolemia; CHF: congestive heart failure; AF: atrial fibrillation; AKI: acute kidney injury; RRT: renal replacement therapy

## Discussion

This observational retrospective study based on a large sample cohort analyzed the relationship between admission WBCc in AAD patients and their clinical outcomes. Our results indicated that a high-WBCc on admission in patients with AAD was associated with poor clinical outcomes. After adjustment using a multivariate Cox analysis, the WBCc is an independent predictor to 30-day, 90-day and 1-year mortality. AUC analysis indicated that the WBCc had a better performance than SIRS score in predicting mortality in patients with AAD. Moreover, a subgroup analysis showed that high-WBCc on admission carried an excess risk of 30-days mortality in patients who were younger than 69 years of age or had a history of respiratory disease.

AAD is an acutely presenting, severe disease and its simple and effective biomarker for evaluating the prognosis is still lacking [14]. Inflammation promotes medial degradation of aortic artery and increases artery wall weakness and rupture [15, 16]. In recent decades, studies showed that several indicators of the inflammatory reaction including CRP level [17], D-dimer level [18] and PLTc [19] were associated with clinical outcomes in acute aortic syndrome (AAS). The WBCc is a sensitive and non-specific inflammation biomarker and its elevation also has been observed in AAD patients [11, 12, 20, 21]. However, the results of further studies on the association between the WBCc and prognosis of patients with AAD were inconsistent. A French study [20] with a Western cohort showed that the WBCc was not associated with in-hospital death in patients with AD (OR = 2.80, 95% CI 0.80–12.58, *P* = 0.12), but its sample size was relatively small (n = 94). Recently, two studies from China [11, 12] respectively found that, the WBCc could predict in-hospital, but failed to post-discharge mortality. The differences in underlying diseases, type of AD and sample quantity may partially explain the inconsistency.

In the present study, approximately a quarter of AAD patients showed an elevated WBCc on admission. These patients had higher PLT, HCT, Hb, BUN and Glucose, and had more CHF, higher SAPS II and SIRS scores. Moreover, a novel finding in our study was that the admission WBCc could predict post-discharge including 30-day, 90-day and 1-year mortality of AAD patients, which has rarely been reported before. The results from subgroup analysis and AUC analysis proved an excellent performance of the WBCc in predicting mortality in different periods in AAD patients. Compared with other classic severity scores, the WBCc showed a better performance than SIRS score. The progression of AAD significantly increases the level of vascular inflammation and the White blood cell (WBC) is an inflammatory reactant in the early stage of AAD. It has been proved that WBC can activate endothelial and microvascular damage, resulting in release of pro-inflammatory cytokines that contribute to a profound degradation of collagen and the extracellular matrix (ECM) related to smooth muscle cell (SMC) depletion, elastic fiber fragmentation and atherosclerosis underling aortic wall irreversible remodeling and weakness, which promote the progression of AAD and its postoperative recurrence [15]. In addition, clinical studies showed that an increased WBCc on admission was related to some serious postoperative complications, such as sepsis, hemorrhage, delirium, stroke and myocardial infarction, which might be one of the reasons for the poor prognosis and death [22–25]. In this study, a definite trend is evident that the incidence of malperfusion (including myocardial infarction, ischemic stroke and acute renal injury) in the high-WBCc group were higher than those in the normal-WBCc group, although there was no statistical difference (*P* = 0.094; *P* = 0.683; *P* = 0.264, respectively). Besides, the deaths in both groups were due to proximal or distal extension of dissection, aortic rupture, hemorrhage, pericardial tamponade, myocardial infarction, arch vessel occlusion causing stroke, visceral ischemia. These results are consistent with previous studies and suggests that the elevated WBC may affect clinical outcomes by promoting the progression of AAD and increasing complications. What’s more, our study showed that the mortality rates at 30-day, 90-day, 1-year and 5-year in high-WBCc group were significantly higher than those in the normal-WBCc group (*P* = 0.003; *P* < 0.001; *P* < 0.001; *P* = 0.006, respectively), but Kaplan Meyer survival curve indicated that the main drop in high-WBCc group was concentrated at the beginning of the curve. This is most likely due to the severity of the disease including malperfusion or comorbidities (e.g. CHF) in these patients as mentioned above. After initial drop, the survival curves in two groups didn’t separate further. This result suggested that admission WBCc were more associated with 30-day, 90-day and 1-year outcomes, which was also shown in the Cox regression analysis.

In the subgroup analysis, there was no interaction in most strata, which proved the reliability of the WBCc on admission predicting mortality in AAD patients. We also found that AAD patients who were younger than 69 years of age or had a history of respiratory disease with an elevated WBCc had an excess risk of 30-days mortality. On the one hand, we analyzed the data of major organ perfusion in young patients with the high-WBCc and elderly patients with high-WBCc. A definite trend is evident that the incidence of acute renal injury in the young patients were higher than elderly patients (50.0% vs. 41.2%, *P* = 0.434). It has long been known that malperfusion such as acute kidney injury was an independent predictor of mortality in AAD [26, 27]. Although there is no statistical difference in this result, it suggested that the higher risk of 30-day mortality in younger patients with high-WBCc may be associated with this complication, which need to be confirmed in a larger population. On the other hand, postoperative hypoxemia is quite common in patients with AAD and is associated with poor clinical outcomes [28]. Previous studies have shown that AAD patients complicated with respiratory diseases such as chronic obstructive pulmonary disease (COPD) are more likely to have hypoxemia or develop into acute respiratory failure after surgery, which has been proved to be an independent predictor of mortality in AAD [29, 30]. Besides, patients with respiratory diseases such as COPD, asthma and tuberculosis accompanied with an increased WBCc have a higher mortality rate [31, 32]. Perhaps, it could explain the reason why AAD patients who had a history of respiratory disease with an elevated WBCc had an excess risk of 30-days mortality. Our results indicated that more severe measures need to be taken in both of the above situations.

It is first time to reveal the potential value of the WBCc as a prognostic biomarker of post-discharge mortality in AAD patients. Combined with previous studies, our results provide further evidence of the utility of this stable and convenient indicator predicting prognosis in AAD patients. In the future, additional researches are needed to further understand the role of different types of WBC or some of their components in the prognosis of AAD patients, which provide the possibility for the application of targeted intervention in the treatment of AAD.

There are several limitations need to be mentioned in the study. Firstly, this study is a single-center observation research, which may not be universally representative. Secondly, we only focus on the admission WBCc. Observation of changes of the WBCc at different periods may provide more information in assessing its prognostic value. Thirdly, there are some missing data in this study, such as the timing of admission from onset, the severity of dissection and the surgical factors, which could also affect the WBCc. More on the above indicators evaluation and in-depth mechanism exploration should be conducted in the future.

## Conclusions

In summary, the present study indicated that higher than normal WBCc on admission is an independent predictor for the post-discharge mortality in patients with AAD.

## Data Availability

The datasets generated and/or analysed during the current study are available in the Physionet repository, https://physionet.org/physiobank /database/mimic 3cdb.

## Abbreviations

AAD: Acute aortic dissection
AD: Aortic dissection
WBCc: White blood cell count
ROC: Receiver operating characteristic
ICU: Intensive care unit
CRP: C-reactive protein
PLTc: Platelet count
MIMIC: Medical Information Mart for Intensive Care
BIDMC: Beth Israel Deaconess Medical Center
HIPAA: Health Insurance Portability and Accountability Act of 1996
ICD-9: International Classification of Diseases 9th Edition
SQL: Structured Query Language
SBP: Systolic blood pressure
DBP: Diastolic blood pressure
MBP: Mean blood pressure
RBC: Red blood cell
RDW: Red cell distribution width
HB: Hemoglobin
HCT: Hematocrit
PLT: Platelet
APTT: Activated partial thromboplastin time
PT: Prothrombin time
INR: International normalized ratio
BUN: Blood urea nitrogen
Cr: Creatinine
HC: Hypercholesterolemia
CAD: Coronary artery disease
CHF: Congestive heart failure
AF: Atrial fibrillation
AKI: Acute kidney injury
RRT: Renal replacement therapy
SOFA: Sequential organ failure assessment
SAPS II: Simplified acute physiology score
OASIS: Oxford acute severity of illness score
SIRS: Systemic inflammatory response syndrome
IQR: Interquartile range
HRs: Hazard ratios
CIs: Confidence intervals
AAS: Acute aortic syndrome
WBC: White blood cell
ECM: Extracellular matrix
SMC: Smooth muscle cell
COPD: Chronic obstructive pulmonary disease
